# The Almost Perfect Scale in medical students: factor analysis, measurement invariance, and profile analysis

**DOI:** 10.3389/fpsyg.2023.1188187

**Published:** 2023-07-14

**Authors:** Elizabeth H. Ellinas, Tavinder K. Ark, Catherine C. Ferguson, Bo Zhang

**Affiliations:** ^1^Data Science Lab, Robert D. and Patricia E. Kern Institute for the Transformation of Medical Education, Center for the Advancement of Women in Science and Medicine, Department of Anesthesiology, Medical College of Wisconsin, Milwaukee, WI, United States; ^2^Data Science Lab, Robert D. and Patricia E. Kern Institute for the Transformation of Medical Education, Medical College of Wisconsin, Milwaukee, WI, United States; ^3^Robert D. and Patricia E. Kern Institute for the Transformation of Medical Education, Department of Emergency Medicine, Medical College of Wisconsin, Milwaukee, WI, United States; ^4^Department of Educational Psychology, University of Wisconsin–Milwaukee, Milwaukee, WI, United States

**Keywords:** differential item functioning, factor analysis, latent profile analysis, medical students, perfectionism

## Abstract

Incoming medical students at a private midwestern medical school are routinely surveyed at the time of matriculation on wellness measures, one of which is the Almost Perfect Scale – Revised (APS-R). An 8-item subset of this 23-item scale has been suggested as an alternative perfectionism measure, called the Short Almost Perfect Scale (SAPS). To confirm the within-network and between-network construct validity of both scales in our population, responses in 592 matriculating medical students from the years 2020–2022 were analyzed using both versions of this scale. Confirmatory factor analysis found the items significantly measured the construct of perfectionism in the SAPS scale, but not the APS-R. The APS-R was not analyzed further. SAPS was analyzed for measurement invariance (MI) and was equivocal for gender at the scalar level; differential item functioning indicated that any MI effect was small. Latent profile analysis was inconclusive in our sample, possibly because our students’ scores on the latent variable “standards” were consistently higher than previously reported. We recommend that the SAPS be used rather than the APS in medical students, that gender differences be analyzed with caution, and that profiles of types of perfectionists not be utilized in this population without further investigation. Finally, we suggest that the discrepancy scale alone may be a better indicator of perfectionism in this population of high achievers.

## Introduction

Medical student wellness has been a concern for medical schools, with over 85,000 papers on the topic available through the search engine “PubMed” as of December 2022, and 20,000 of those in 2021–2022 alone. Concerns specifically regarding mental health of medical students have led to discovery of high rates of anxiety (Quek et al., 2019) and depression (Rotenstein et al., 2016; Blacker et al., 2019) in students, along with stigma against asking for mental health assistance (Hankir et al., 2014; Blacker et al., 2019). Seeking both causes and solutions for mental health concerns, many have turned to underlying traits of students, employing scales to identify those at risk.

One such trait, perfectionism, is “a personality style characterized by striving for flawlessness and setting of excessively high standards for performance accompanied by tendencies for overly critical evaluations of one’s behavior” (Stoeber and Otto, 2006, p. 295). Perfectionism is particularly likely to be present in medical students, who must embody excellence to be accepted into medical school, and then are subject to both high performance standards and abundant opportunities for self-critical comparison to outstanding peers. Perfectionism was proposed as a factor with both positive and negative elements as early as the 1970’s (Hamachek, 1978), with most of the focus on its association with psychopathology. In order to quantify amounts of perfectionism, several scales have been developed, including the Almost Perfect Scale (APS) (Slaney et al., 1995). The most common version of this scale, the APS-Revised or APS-R (Slaney et al., 2001), has items for both positive or adaptive perfectionism (having high personal “Standards” for oneself) and maladaptive perfectionism (feeling a “Discrepancy” between personal standards and performance).

Meta analysis indicates a strong relationship between depression and perfectionistic concerns in non-medical students, with a weighted averaged zero-order effect size of 0.40 (Limburg et al., 2017), and in medical students, maladaptive perfectionism has been significantly associated with shame and embarrassment, which are in turn associated with depression, anxiety, and suicidal ideation (Enns et al., 2001; Brennan-Wydra et al., 2021). In a study by Hu and colleagues, more than 60% of medical-student participants compared their academic performance to others at least moderately, and two-thirds reported tying their academic performance to their self-worth (Hu et al., 2019). These findings suggest that a focus on perfection and concerns over mistakes may lead to poor coping with medical errors (Robertson and Long, 2018; Leung et al., 2019).

The APS has two versions: the APS-R, and the Short Almost Perfect Scale (SAPS). Given their extensive use and relationship to devastating outcomes, we examine the within-network and between-network construct validity evidence for perfectionism scales in medical students (American Educational Research Association, 2014).

## Materials and methods

### Study design

The institutional review board approved this cross-sectional, observational study using a convenience sample of medical students. Beginning with within-network validity, we first utilize confirmatory factor analysis (CFA) to determine the factor structure of the APS-R and SAPS in medical students and provide evidence regarding whether APS-R or SAPS can be used to measure perfectionism in this population. Second, as gender has been found to be an important factor in medical student depression and wellness (Dahlin et al., 2005; Fukushima et al., 2020), we utilize measurement invariance (MI) to evaluate whether the APS measures perfectionism equivalently for men and women students. And, if MI is violated, differential item functioning (DIF) is conducted to identify specific items that may contribute to the violation. Third, the APS developers created cut offs to describe three distinct groups of perfectionists: non-perfectionists, adaptive perfectionists, and maladaptive perfectionists. Through our analyses, we hoped to replicate the patterns of perfectionism previously found through latent profile analysis (LPA) (Rice and Ashby, 2007; Rice et al., 2014) to validate the author cut-offs (group ownership). To determine between-network validity, we examine APS’s relationship to external variables, providing evidence that the APS scales are appropriately associated with other theoretically related scales, and utilizing other constructs including depression, self-compassion, and coping.

### Participants

All 793 students entering their first year of medical school at a private midwestern university during the 3 years 2020–2022 were included in a voluntary survey which consisted of various assessment measures as part of a wellness curriculum. One of these measures was the APS-R. Participation was voluntary and completed online during class time. Six-hundred-nineteen (78%) responded to the survey. Four students listed their gender as non-binary and were subsequently removed from the quantitative analysis due to small group size. Of the remaining students, 592 selected a listed gender and completed all items on the APS-R, representing 74.7% of the three incoming medical school classes [232/264 (87.8%) from 2020, 181/263 (68.8%) from 2021, and 179/266 (67.3%) from 2022]. Of these, 334 (56%) self-identified as women, and 258 (44%) identified as men, consistent with the genders of the matriculants from those 3 years (57.8% women, 42.2% men).

### Instruments

The APS-R (Slaney et al., 2001) has a total of 23 items: 4 items for orderliness (usually ignored; Stoeber and Otto, 2006), 7 items for adaptive perfectionism or “standards” (e.g., I expect the best from myself.), and 12 items for maladaptive perfectionism or “discrepancy” (e.g.,” my performance rarely measures up to my standards). Each item uses a 7-point Likert scale: strongly disagree (1) to strongly agree (7). APS-R scoring classifies respondents into three groups: non-perfectionists, adaptive perfectionists, and maladaptive perfectionists (Rice and Ashby, 2007). Classification is based on the total score from the 19 standards and discrepancy items. Recommended APS-R scoring follows the following steps: sum the items for standards (maximum possible 49). If your standards score sum <42 the respondent is a non-perfectionist. If the standards score > =42, check the discrepancy score (maximum possible 84). If the discrepancy score < 42 the respondent is an adaptive perfectionist. If the discrepancy score is > = 42 the respondent is a maladaptive perfectionist.

Rice and colleagues (Rice et al., 2014) proposed an 8-item “short form” for the APS-R called the SAPS. SAPS utilizes items directly taken from the APS-R and consists of four questions each on standards and discrepancy. Rice’s stated goals for the SAPS were to reduce redundancy and ambiguity while creating a shorter but equally psychometrically valid perfectionism scale. Although the APS-R continues in use, since its introduction in 2014, SAPS has gained in acceptance, has been translated into several languages (de Holanda et al., 2021), and at least one attempt has been made to classify perfectionists using this scale (Wang et al., 2016).

### Statistical analysis

Analyses were conducted in R (The R Core Team, 2021), using LAVAAN for CFA and MI (Rosseel, 2012), lordif (Choi et al., 2011) for differential item functioning, and MCLUST (Scrucca et al., 2016) for LPA. To assess within-construct validity, the internal structure of the scales was tested using CFA. CFA was conducted using Robust Maximum Likelihood (RML) estimation because the standards items exhibited significant skewness. Schreiber’s recommendations (Schreiber, 2017) were utilized to determine model fit using the following indicators: chi-squared (χ^2^) ratio to degrees of freedom (df) ≤ 2–3, root-mean-squared error of approximation (RMSEA) <0.05, comparative fit index (CFI) ≥ 0.95, Tucker-Lewis index (TLI) ≥0.95, and Standard Root Mean Residual (SRMR) ≤0.08. Measurement invariance was conducted *via* multi-group CFA using the Santorra-Bentler method to compensate for non-normality. Chi-square difference tests (*p* < 0.05), change in CFI and RMSEA were conducted to determine if significant changes in model fit existed for nested models (e.g., concurrently constraining parameters) for measurement equivalence. Most previous research on the APS-R and SAPS scales utilizes MPLUS (Muthén and Muthén, 1998-2017) for LPA analysis. Per Muthen, (Muthen 2020) MCLUST’s EEI structure (within-class covariance matrix is diagonal, equal volume and shape) duplicates MPLUS most closely, and therefore EEI was chosen as the multivariate mixture model in MCLUST.

For between-construct validity, we selected scales with known association to perfectionism, examining whether the APS “behaves as it should” within this population. The Depression Anxiety Stress Scale (DASS) (total summed score) was selected because of depression’s known positive association with both adaptive and maladaptive perfectionism (Limburg et al., 2017; Ferrari et al., 2018; Liu et al., 2022). As there are many scales for depression and perfectionism, the suggested correlations are non-specific, often relying upon studies that utilized different scales for depression and perfectionism, but nonetheless confirming a connection between the two. For example, Limburg, in meta-analysis, found a correlation of 0.45 (*p* < 0.001) between maladaptive perfectionism (which they termed “perfectionistic concerns”) and depression, and also a correlation of 0.18 (*p* < 0.05) between adaptive perfectionism and depression (Limburg et al., 2017). For discriminant validity, the Self-Compassion Scale (SCS) and Brief COPE scale (BCS) are selected because of emerging evidence that self-compassion and coping skills influence the effects of on psychopathology. For SCS, the total scale score was used (Neff, 2023); for BCS, the scale’s originators do not recommend a total score be used; we elected to use mean “approach” and “avoid” subscales (Eisenberg et al., 2012). While many fewer studies exist than for depression, we expect maladaptive perfectionism would have a substantial negative correlation with SCS (Ferrari et al., 2018; Richardson et al., 2020; Wei et al., 2021), a substantial positive correlation with BCS avoid, and no correlation with BCS approach (Fye et al., 2018; Vanstone and Hicks, 2019; Collin et al., 2020). Studies that included adaptive perfectionism in their analysis are even fewer, but suggest we should expect a small positive correlation with SCS (Wei et al., 2021), no to a small negative correlation with BCS avoid, and a small positive correlation with BCS approach (Fye et al., 2018; Vanstone and Hicks, 2019; Collin et al., 2020).

## Results

The mean standards and discrepancy scores were calculated for the group as a whole and then by gender for the APS-R (the whole scale) and SAPS items (as a subset of responses to the APS-R). Mean (standard deviation) scores for standards were 6.25 (0.65) APS-R and 6.33 (0.75) SAPS. Mean (standard deviation) scores for discrepancy were 3.60 (1.42) APS-R and 3.34 (1.54) for SAPS. Student’s t-test found no significant differences in means by gender for APS-R or SAPS.

### Within-measure validity

#### Confirmatory factor analysis

Previous research supports a two-factor model for APS-R with 7 items measuring the standards factor and 12 items the discrepancy factor (Slaney et al., 2001). Using Schreiber’s recommended fit statistics (Schreiber, 2017), the model fit for APS-R was poor. Of the five tested fit indices, specifically chi-square = 668.377 (df = 151, *p* = 0.000) with a chi-square ratio = 4.4, CFI = 0.920, TLI = 0.909, and RMSEA 0.083, SRMR = 0.052, only SRMR is within Schreiber’s guidelines for good fit. Previous research supports a two-factor model which constrains the 8 SAPS items to load as 4 items for standards and 4 for discrepancy (Rice et al., 2014). CFA confirms this fit in our model for all 5 fit indices: chi-square = 58.817 (df 19, *p* = 0.000), ratio of chi-square to df 3.1, CFI = 0.971, TLI = 0.958, RMSEA 0.059, SRMR 0.033. The factor loadings for both models can be found in Table 1. Finding that the APS-R model did not fit in our population as well for previously published populations, and the SAPS model fit our population well, the SAPS version was deemed the preferable version for our population and utilized in all further analyses.

**Table 1:** Factor loadings in CFA for APS-R and SAPS.

APS-R		SAPS	
Items	Loadings	Items	Loadings
**Standards**		**Standards**	
Standards 1	0.719	Standards 8	0.793
Standards 5	0.255	Standards 12	0.843
Standards 8	0.814	Standards 14	0.749
Standards 12	0.808	Standards 22	0.705
Standards 14	0.746		
Standards 18	0.541		
Standards 22	0.726		
**Discrepancy**		**Discrepancy**	
Discrepancy 3	0.508	Discrepancy 11	0.851
Discrepancy 6	0.777	Discrepancy 16	0.859
Discrepancy 9	0.762	Discrepancy 20	0.868
Discrepancy 11	0.874	Discrepancy 23	0.729
Discrepancy 13	0.840	Covariance = 0.112	
Discrepancy 15	0.643		
Discrepancy 16	0.852		
Discrepancy 17	0.757		
Discrepancy 19	0.861		
Discrepancy 20	0.885		
Discrepancy 21	0.865		
Discrepancy 23	0.733		
Covariance = 0.107			

APS-R, almost perfect score – revised. SAPS, short almost perfect score. All loadings significant at the *p* = 0.000 level.

#### Measurement invariance

Table 2 presents the results of the MI analysis by gender for SAPS, and includes both the chi-square parameters, and the chi-square difference between successive MI nested models. Results show that the SAPS model was invariant to gender at the configural and metric levels, with RMSEA and SRMR not substantially increasing, and CFI, TLI and chi-square change not substantially decreasing as the model constraints were sequentially imposed. Thus, results suggest that the pattern of loadings and loadings themselves were equivalent by gender. However, utilizing chi-square as criteria, the SAPS model failed at the scalar (intercept) level (*p* = 0.006). Authors have suggested cut-off scores for other criteria, including ΔCFI ranging from <0.01 to <0.007 and ΔRMSEA <0.01 to indicate invariance (Rutkowski and Svetina, 2017; Lee and Smith, 2020). Utilizing these criteria, MI was equivocal at the scalar level with ΔRMSEA = 0.002 and ΔCFI = 0.005.

**Table 2:** Measurement invariance for the Short Almost Perfect Scale (SAPS).

	ChiSq	df	ChiSq/df	CFI	RMSEA	TLI	SRMR	ChiSq Δ	df Δ	p ChiSqΔ	RMSEA Δ	CFI Δ	TLI Δ
Configural (Structure)	88.493	38	2.329	0.981	0.065	0.972	0.033						
Metric (Loadings)	100.565	44	2.286	0.978	0.065	0.971	0.043	12.427	6	0.053	0.000	−0.003	0.000
Scalar (Intercepts)	118.606	50	2.372	0.973	0.067	0.970	0.045	18.156	6	**0.006**	0.002	−0.005	−0.002

df, degrees of freedom; RMSEA, root mean square error of approximation; SRMR, standardized root mean square residual; CFI, comparative fit index; TLI, Tucker–Lewis Index; ChiSq, chi-square; diff, difference; bolded *p* < 0.05, Scaled Chi-Squared Difference Test (method = Santorra-Bentler).

#### Differential item functioning

Having achieved mixed results for scalar invariance, DIF was utilized to determine which items might have contributed to the variance between men and women on the SAPS. “Uniform DIF” represents a discrepancy between the conditional and grouping models, “non-uniform DIF” a discrepancy between the grouping and interaction models, and “total DIF effect” guards against type 1 error (in large samples) and should be significant if either uniform or non-uniform DIF is present. Table 3 presents the DIF results from ordinal logistic regression analysis of SAPS items. Testing was completed for the standards items separately from the discrepancy items. Using an alpha level of 0.05, two items were flagged for DIF, standards item 8, “I have high expectations for myself” and discrepancy item 20, “I am hardly ever satisfied with my performance.” Standards item 8 failed to reach significance at the “total DIF” level and is therefore possibly type 1 error. Discrepancy item 20 exhibited elements of uniform and non-uniform DIF, consistent with concerns at both the metric and scalar levels. However, McFadden’s *R*^2^ is less than 0.013, indicating that although DIF is present, its effect is small (Jeong and Lee, 2016).

**Table 3:** Differential item functioning (DIF) for the Short Almost Perfect Scale (SAPS).

Item	#cat	Uniform DIF	Non-uniform DIF	Total DIF effect	DIF present	*R* ^2^
Standards 8	3	**0.002**	**0.009**	0.967	NO	0.009
Standards 12	4	0.598	0.204	0.088		0.000
Standards 14	4	0.563	0.566	0.370		0.000
Standards 22	4	0.713	0.908	0.810		0.000
Discrepancy 11	7	0.424	0.712	0.840		0.000
Discrepancy 16	7	0.414	0.716	0.956		0.000
Discrepancy 20	7	**0.000**	**0.000**	**0.038**	YES	0.008
Discrepancy 23	7	0.930	0.811	0.521		0.000

Item labels indicate the item category (standards or discrepancy) and the number of the item in the APS scale. #cat, number of categories for that item after collapsing cells with < 5 entries; *R*^2^, McFadden’s effect size, bolded *p* < 0.05.

#### Latent profile analysis

Table 4 attempts to replicate the findings of Rice et al. (2014), using model specifications equivalent to the EEI setting in Mplus in MCLUST (Muthen, 2020). The fit indices each recommended different models as the best fit; that is, there was no consensus between the various model fit indices in selecting one preferred model. The Bayesian Information Criterion (BIC) indicated that the preferred model had at least eight classes, which would likely be uninterpretable based on previous work with perfectionism. All models had entropy ≥0.08 (Wang et al., 2017), and the highest entropy (0.9694) indicated that a two-class solution might be preferred. This model seems reasonable if medical school matriculants do not have (or have very few) non-perfectionists. Bootstrapped Likelihood Ratio Test was calculated using tidyLPA (Rosenberg et al., 2019) and is inconclusive: the consistent *p* < 0.05 for each class comparison indicates that each successive group is significantly better than the c-1 model. Finally, one-way analysis of variance (ANOVA) was conducted on the SAPS means with the different latent-class SAPS groups as the between subjects factor. *Post hoc* Tukey tests were conducted with the group closest to representing adaptive perfectionists (high standards mean, low discrepancy mean; Rice et al., 2014) utilized as the comparison group. Significant differences in SAPS mean scores were found between the classes in both the 2-class and 3-class model. Post-Hoc Tukey test found classes that corresponded to previously found class characteristics, including adaptive perfectionism (high standards, low discrepancy), maladaptive perfectionism (high standards, high discrepancy), and in the three-class model, non-perfectionism (low standards, low discrepancy). The addition of a fourth class added another group that appeared to overlap with adaptive perfectionists, and adding a fifth class produced a small group (24 people) not typically, but occasionally found in other studies: low standards, high discrepancy (Wang et al., 2007).

**Table 4:** Fit indices for latent profile analysis using MCLUST’s EEI structure (within-class covariance matrix is diagonal, volume and shape are held equal) to best replicate results in MPLUS.

Classes and counts				Fit indices				Difference in means (ANOVA)			Potential class labels
Model	Classes	Class proportion	Class count	BIC	Entropy	BLRT	BLRT p	Standards	Discrepancy	Eta	
1	1	1.00	592	15677.4	1.000	NA	NA				
2	1	0.30	179	14440.0	**0.969**	1294.89	0.010	***0.26	***2.83	stand 0.025 disc 0.717	Maladaptive
	2	0.70	413					0.00	0.00		Adaptive
3	1	0.26	157	13748.8	0.948	748.62	0.010	0.05	***3.06	stand 0.612 disc 0.698	Maladaptive
	2	0.51	300					0.00	0.00		Adaptive
	3	0.23	135					***–1.38	***0.72		Non-perfection
4	1	0.19	112	13628.9	0.921	177.34	0.010	*0.14	***3.72	stand 0.594 disc 0.811	Maladaptive
	2	0.45	265					0.00	0.00		Adaptive
	3	0.18	109					***–1.42	***1.05		Non-perfection
	4	0.18	106					*0.15	***1.8		^Adaptive2
5^**$**^	1	0.20	118	13288.2	0.943	398.13	0.010	−0.13	***3.7	stand 0.731 disc 0.816	Maladaptive
	2	0.33	196					0.00	0.00		Adaptive
	3	0.04	24					***–2.48	***2.04		Low standards, high discrepancy
	4	0.27	159					***–1.12	***0.54		Non-perfection
	5	0.16	95					0.03	***1.81		^Adaptive2
6	6			13070.5	0.964	275.19	0.010				
7	7			12970.4	0.950	157.54	0.010				
8	8			**12955.1**	0.930	72.76	0.010				

For all models, the group that best replicated adaptive perfectionists found in previous published studies (high standards and low discrepancy) were set as the comparison class (mean referenced as 0.00), and means for other classes are relative to the comparison class. *p < 0.05; ***p < 0.001; BIC, Bayesian information criterion; BLRT, bootstrapped likelihood ratio test: BLRT and BLRT p are from Tidy LPA; bolded = best fit for that indicator; ^adaptive2 = a potential second group that fit criteria for adaptive perfectionists (high standards, low discrepancy). ^**$**^We did not calculate fit indices for groups > 5 because with groups = 5, one group had only 24 respondents in it.

Given these indeterminate results, we considered other models using MCLUST, which allows a multivariate analysis in which the volume, shape, and orientation of the covariances can all be constrained or allowed to vary among classes, producing 14 potential models. None of these models had better fit than EEI.

### Between-measures validity

#### Correlations to external scales

We found an unexpected non-correlation between standards and DASS (*r* = 0.069, *p* = 0.094) and small significant correlations between standards and self-compassion (*r* = −0.104, *p* = 0.016) and BCS avoid (*r* = 0.095, *p* = 0.021) and BCS approach (r = 0.155, *p* = 0.000). There were expected moderate to large correlations between discrepancy and DASS (*r* = 0.537, *p* = 0.000) consistent with convergent validity, moderate to large negative correlations between discrepancy and SCS (*r* = −0.521, *p* = 0.000), and BCS-avoid (*r* = 0.453, *p* = 0.000), consistent with discriminant validity, and no correlation to BCS-approach (*r* = −0.009, *p* = 0.826).

## Discussion

This study of 592 matriculating medical students, attempted to validate two perfectionism scales: the APS-R and SAPS. Within-measure construct validity findings validated the internal structure of the SAPS but not APS-R, found equivocal measurement non-invariance by gender, and did not find previously published latent profile patterns. Between-measures construct validity was found for discrepancy but not standards. In an examination of internal structure, CFA for the APS-R failed in our medical student population, suggesting that medical school responses to the APS-R do not measure the construct of perfectionism as it did in other populations from previous studies (Slaney et al., 2001; Rice et al., 2014). Examination of the SAPS indicated that a two-factor model held for medical students, and this scale was utilized for all subsequent examinations. This finding in favor of SAPS is consistent with findings in other populations (Rice et al., 2014; Kira et al., 2018; de Holanda et al., 2021), and suggests that the SAPS is likely preferred in the medical student population.

The SAPS was invariant by gender up to the metric level but questionable at the scalar level, although subsequent DIF examination indicated that was any effect is likely small. Most validity studies of the APS have been completed in convenience samples of university students in psychology classes, which resulted in female-predominant samples. MI results have been mixed by gender, with most studies supporting invariance (Rice et al., 2014; Kira et al., 2018; de Holanda et al., 2021), while a few have indicated at least partial non-invariance at the scalar (intercept) level (Rice et al., 2019). In our population, indeterminate measurement invariance results suggest that any measured mean differences in perfectionism by gender may result from the ways groups perceive perfectionism or reacted to the scale questions, rather than actual differences in the mean levels of perfectionism, and that future work is needed to confirm MI by gender in this population.

We used LPA to seek perfectionism profiles within our student population, hoping that those profiles could give us deeper insights and allow us to categorize our student populations into at-risk groups. We were not able to find the same three-profile pattern in this population of medical students as the authors of the scale found in other populations, and regrettably were not able to indicate any distinct profiles, instead finding that each index seemed to favor a different number of classes. While most authors have concluded that a three-class model was appropriate (Rice and Ashby, 2007; Rice et al., 2014), Wang et al. (2007) found a fourth class – one with low standards and high discrepancy – that they suggested might be a result of external pressures such as parental expectations. It’s additionally possible that our uniquely perfectionistic population had nearly a two-class model without non-perfectionists, which kept us from finding the typical profile classes. Finally, Flett et al. (2016) suggested that discrepancy could be multidimensional, containing elements of both dissatisfaction and falling short of expectations, a situation that seems possible in the high-stress, high-stakes medical environment, and might be affecting our profile allocations.

Finally, we sought between-measures validity evidence by correlating standards and discrepancy subscales with other constructs, finding that the discrepancy scale performed better than standards on these measures. For discrepancy, moderate to large correlations between discrepancy and depression were found as historically expected (Limburg et al., 2017; Liu et al., 2022). Additionally, negative correlation to self-compassion, positive correlation to BCS avoid and no correlation to BCS approach suggest that discrepancy is tracking well with new data indicating a connection between self-compassion, coping, and perfectionism (Ferrari et al., 2018; Fye et al., 2018; Vanstone and Hicks, 2019; Collin et al., 2020; Richardson et al., 2020; Wei et al., 2021). Unexpectedly, the standards subscale – which typically also correlates with depression but not as strongly as discrepancy (Limburg et al., 2017) – did not correlate with depression, and additionally unexpectedly was negatively correlated with self-compassion and positively correlated with negative coping (BCS-avoid). The only expected correlation for standards was a small positive correlation with BCS-approach (Fye et al., 2018; Vanstone and Hicks, 2019; Collin et al., 2020; Wei et al., 2021). Overall, this leads us to conclude that the APS discrepancy scale “behaves correctly” with other scales, while standards exhibits small, possibly not meaningful, correlations in unexpected directions, suggesting that discrepancy is a better measure in our population than standards.

Relative to the general population, our medical students showed similar SAPS scores for discrepancy [mean 3.34 (1.54)], but higher and more consistent scores on standards 6.33 (0.75) – typical scores in non-medical students being closer to 6.0 (Rice and Ashby, 2007). Moreover, the LPA entropy results (somewhat pointing to but not conclusive for a two-class solution) suggest that perhaps most medical students are perfectionists, and what distinguishes groups is the discrepancy score. Maladaptive perfectionism by itself is a strong predictor of depression (Limburg et al., 2017; Seeliger and Harendza, 2017), and self-compassion and coping strategies may mediate the relationship between perfectionism and depression (Ferrari et al., 2018; Collin et al., 2020; Wei et al., 2021), suggesting that the discrepancy score alone may be a better indicator of perfectionism in our population and suggesting a direction for future work.

### Limitations

This study has several limitations. First, all students took the 23-item APS-R (including the four Order items). For the SAPS scale analysis, the authors selectively analyzed the SAPS items. As context matters – responses on individual items influence choices on adjacent items (Şahin, 2021) – future iterations should include a group of students that takes only the 8-item SAPS. Second, this work represents first-year medical students in a single, private medical institution in the Midwest United States; therefore, caution should be used if generalizing these findings to other medical schools or other years in medical school. Further, this study considered only binary gender, and discarded the responses from the low numbers of students (less than five) sharing a gender that was non-binary. As best practices expand regarding survey items for non-binary gender, guidelines remain elusive regarding statistical best practices for working with those voices, as they are often few within the data set, and we look forward to future developments in this area.

### Implications and conclusion

We recommend that the SAPS be used rather than the APS in medical students, that gender differences be analyzed with caution, and that profiles of types of perfectionists not be utilized in this population without further investigation. Finally, we suggest that further work is needed to determine whether the discrepancy scale alone is a better indicator of perfectionism in this population of high achievers.

## Data availability statement

The datasets presented in this article are not readily available because would need institutional review board approval. Requests to access the datasets should be directed to libby@mcw.edu.

## Ethics statement

The studies involving human participants were reviewed and approved by Medical College of Wisconsin Human Research Protection Program (HRPP). Written informed consent for participation was not required for this study in accordance with the national legislation and the institutional requirements.

## Author contributions

EE contributed to the conception, design, and analysis of data and drafted and revised the manuscript. TA and BZ contributed to the conception, design, and analysis of data. All authors contributed to manuscript revision, and read and approved the submitted version.

## Funding

The authors acknowledge the support of the Kern Institute for the Transformation of Medical Education for their salary support of EE, TA, and CF, and for providing access to the wellness curriculum data, and the Medical College of Wisconsin Center for the Advancement of Women in Science and Medicine for the salary support for EE.

## Conflict of interest

The authors declare that the research was conducted in the absence of any commercial or financial relationships that could be construed as a potential conflict of interest.

## Publisher’s note

All claims expressed in this article are solely those of the authors and do not necessarily represent those of their affiliated organizations, or those of the publisher, the editors and the reviewers. Any product that may be evaluated in this article, or claim that may be made by its manufacturer, is not guaranteed or endorsed by the publisher.
